# Movement patterns of the grey field slug (*Deroceras reticulatum*) in an arable field

**DOI:** 10.1038/s41598-020-74643-3

**Published:** 2020-10-21

**Authors:** John Ellis, Natalia Petrovskaya, Emily Forbes, Keith F. A. Walters, Sergei Petrovskii

**Affiliations:** 1 School of Mathematics, University of Birmingham, Birmingham, UK; 2 Centre for Integrated Pest Management, Harper Adams University, Newport, UK; 3 School of Mathematics and Actuarial Science, University of Leicester, Leicester, UK

**Keywords:** Agroecology, Agroecology, Animal behaviour

## Abstract

We report the results of an experiment on radio-tracking of individual grey field slugs in an arable field and associated data modelling designed to investigate the effect of slug population density in their movement. Slugs were collected in a commercial winter wheat field in which a 5x6 trapping grid had been established with 2m distance between traps. The slugs were taken to the laboratory, radio-tagged using a recently developed procedure, and following a recovery period released into the same field. Seventeen tagged slugs were released singly (sparse release) on the same grid node on which they had been caught. Eleven tagged slugs were released as a group (dense release). Each of the slugs was radio-tracked for approximately 10 h during which their position was recorded ten times. The tracking data were analysed using the Correlated Random Walk framework. The analysis revealed that all components of slug movement (mean speed, turning angles and movement/resting times) were significantly different between the two treatments. On average, the slugs released as a group disperse more slowly than slugs released individually and their turning angle has a clear anticlockwise bias. The results clearly suggest that population density is a factor regulating slug movement.

## Introduction

Understanding animal movement on different spatial and temporal scales is a major focus in biology^1–3^. On large scales, animal movement in response to environmental conditions (e.g. habitat loss, depletion of resources or seasonal changes) through dispersal and migration is an important component of the species survival, fitness and geographical distribution^4–7^. On small scales, the reproductive success and the population growth depend on the efficiency of searching for food, shelter and mating partners^8–10^. For these reasons, patterns of animal foraging have attracted considerable attention during the last few decades^3,11–16^. Understanding movement behaviour of invertebrates is important for many practical reasons, in particular because species such as slugs can cause significant damage to crops^17,18^. There is theoretical and empirical evidence that the pattern of individual movement is a factor influencing population abundance over space and time^19–23^. This has a variety of implications in ecology and agroecology; in the context of pest control the knowledge of the pest population’s spatial distribution is important when developing more sustainable control measures^24,25^.

In identifying patterns of individual animal movement, it is particularly important to take account of density dependent responses (if any) which can significantly affect the population spatial distribution. Population density can be a factor resulting in the formation of high density patches by modifying animal movement both on a shorter within-generation time^19^ and on a longer multi-generation time scale^21,26,27^. In this study, we focus on movement of slugs in agricultural environments. Grey field slug is known to often create patches of high population density even in an apparently uniform environment^28^, i.e. in the absence of environmental forcing. Understanding of the mechanisms regulating the patch formation and cohesion is important as it should enhance the development of more targeted, sustainable slug control^24,25,29^. Previous authors have suggested that slug movement could be regulated by the density dependence, so that the movement pattern of an individual slug is different when con-specifics are present nearby^30^. However, the evidence is scarce and often anecdotal rather than documented, partly because of problems relating to tracking of a mollusc that lives both above and below the soil surface^31^.

While data on individual animal movement of mammals, birds and fish are abundant^15,32,33^, movement data for invertebrates such as snails, worms and slugs in their natural environment remain relatively scarce. One reason for that is the small body size, which, given the current development of radio-tag technology, often makes their tagging difficult or even impossible (but see^34^). Other methods of marking are available but methods such as harmonic radar and radio frequency identification (RFID) tagging make it easier to find organisms without having to interfere with their behaviour.

A recent study has developed a technique for implanting RFID tags beneath the body wall of a fully grown grey field slug (*Deroceras reticulatum*), which can be used to follow individual slugs in the field for extended periods of time when they are both on the soil surface and for up to at least 20 cm below the surface^29^. Comparison of tagged and untagged slugs in laboratory tests have shown that after a 14-day post-insertion recovery period, no significant differences occurred between survival rates, egg batch production, food consumption or locomotor behaviour (velocity; distance travelled), enabling the use of the technique for tracking their movement. The method has subsequently been used to follow individuals in wheat crops for extended periods of time, showing that most slugs ($\sim 80$%) forage within a limited area. For example, 5 weeks after release in the field, the mean overall distance from their release point in work conducted in spring was $78.7 \pm 33.7$ cm, and $101.9 \pm 24.1$ cm in autumn experiments^29^. The observations suggested that this limited locomotor behaviour might promote the stability of the patchy distribution of slugs in arable fields, but further work to determine the underlying behavioural mechanisms was required.

In this paper we report the results of the first study of individual movement of *D. reticulatum* to use a unique combination of field tracking technology, in conjunction with a relevant theoretical framework to support analysis and interpretation of the data collected. The hypothesis that behavioural responses to higher slug densities promote the cohesion (thus stability) of slug patches is tested.

## Materials and methods

The potential for density dependent individual movement to influence slug foraging patterns was investigated by recording the movement of individual slugs above and below the soil surface in an experiment conducted (5–25 November 2016) during the period of higher slug activity in autumn, in a commercial winter wheat field (Shropshire, UK; 52,46001.260 N, −2,34050.140 W). A rectangular $6\times 5$ grid with 2m between adjacent traps was established in the study field, with a single unbaited refuge trap consisting of an upturned terracotta plant pot saucer (18 cm diameter; LBS Horticulture Supplies, Lancashire, UK) set at each node. The spacing of trapping nodes reduced the potential for mutual interference between slugs when later released and tracked. Assessments prior to the experiment indicated that there was a low population of slugs (mean of $<1$ per trap) evenly distributed across the selected study area, reducing the potential for naturally occurring individuals to influence the behaviour of experimental animals. No rainfall was recorded throughout the experiment and low spatial variation in moisture content of the soil was noted across the study area, thus minimizing any differential effects on individual slug movement.

Two slugs were taken from the trap at each grid node (5 November) and placed individually in labelled tubes before being returned to the laboratory and maintained singly in a 250 ml circular plastic rearing container under light/temperature regimes reflecting UK autumn conditions (10:14 light:dark cycle; photophase = $15^\circ$C, scotophase = $10^\circ$C) and at a constant $60\%$ humidity^29^. Lettuce leaves (cv. Romaine) were offered ad libitum to each slug as food and replaced with fresh leaves daily, and all slugs were allowed a 48 h acclimation period before being used in experiments^29^.

Following the acclimation period, a RFID tag with a unique code was implanted into each slug using the previously developed procedure^29^. Slugs were gently anaesthetized by exposing them to a $\hbox {CO}_2$ rich environment for 20 s or until they were fully extended. The needle of an MK165 implanter (Biomark USA) was positioned on the left side about three-quarters of the way along the full length of the body from the anterior end, level with the top of the keel, and with the its tip pointing forward. It was inserted by applying gentle pressure until the tip was no longer visible, and an $8 \times 1$ mm tag (chip and antenna coil encased in glass; HPT8 tag, Biomark USA) was released. Following tagging, each slug was returned to its rearing cage and maintained under the above conditions for the 14 day recovery period required by the procedure. At the end of this period any slug displaying abnormal behaviour (1 individual) was discarded. Only slugs exceeding a minimum weight ($>300$ mg) were tagged. For more details of the procedure, see^29^.

**Figure 1:**
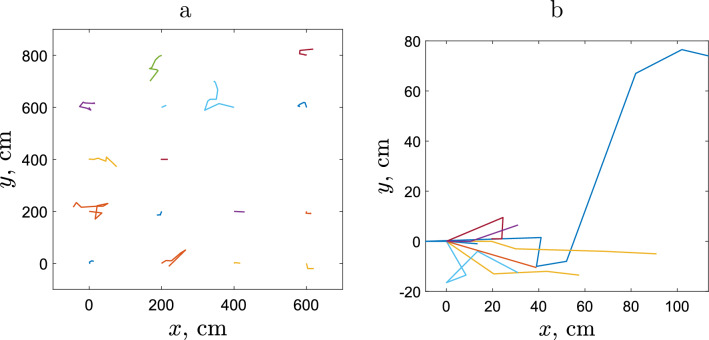
Slug movement paths in an arable field over the total 10 h period of tracking in case of **(a)** sparse release, **(b)** dense release. Note the overall size of the tracking area is much larger in case **(a)** than in case **(b)**.

After the 14 day recovery period, the slugs were allotted at random to one of two treatments before being released back onto the field trapping grid at dusk. Seventeen tagged slugs were each released singly (sparse release), using the nodes on which they had originally been caught to ensure appropriate spacing between slugs and thus minimising mutual contact. In the dense release treatment, eleven tagged slugs were released in close proximity to each other (approximately 20 cm between nearest neighbours). Numbering grid nodes sequentially from the top left corner of the grid matrix of five rows and six columns, the first row contains traps 1–6; in second row the node immediately below node 1 is node 7; et seq. Using this notation, the slugs in the dense release treatment were released on node 18, i.e. approximately in the middle of the area covered by the trapping grid. Movement of each slug in both the sparse and dense release treatments during the following 10 h period, was tracked by mapping its position on a two-dimensional horizontal plane on ten occasions, using a HPR Plus reader and racket antenna (Biomark, USA); see Fig. 1. Both treatments were run simultaneously, and there were no less than 20 min between each assessment although the exact time interval was dependent on the degree of difficulty of locating individuals. The system allowed the position of each slug to be detected whether above or below the soil surface, and the location of each detection was marked using a marker peg and the time of the assessment recorded. To reduce the impact of the accumulation of any minor errors in measurement that may have accrued between sequential markers, the peg locations in relation to the original release point were determined and the distance between sequential markers calculated by triangulation. All slugs were located at each trapping assessment, and combining all assessments slugs were found to be on the soil surface on 79.3% of occasions. Temperature readings taken using a soil temperature probe (Vegetronix, USA) were verified by comparison with the Harper Adams University weather station (situated 650 m from the experimental site on similar open ground and at the same altitude), and fluctuations across the area and during the total time of the experiment never exceeded 3 $^{\circ }$C.

## Analysis and results

**Figure 2:**
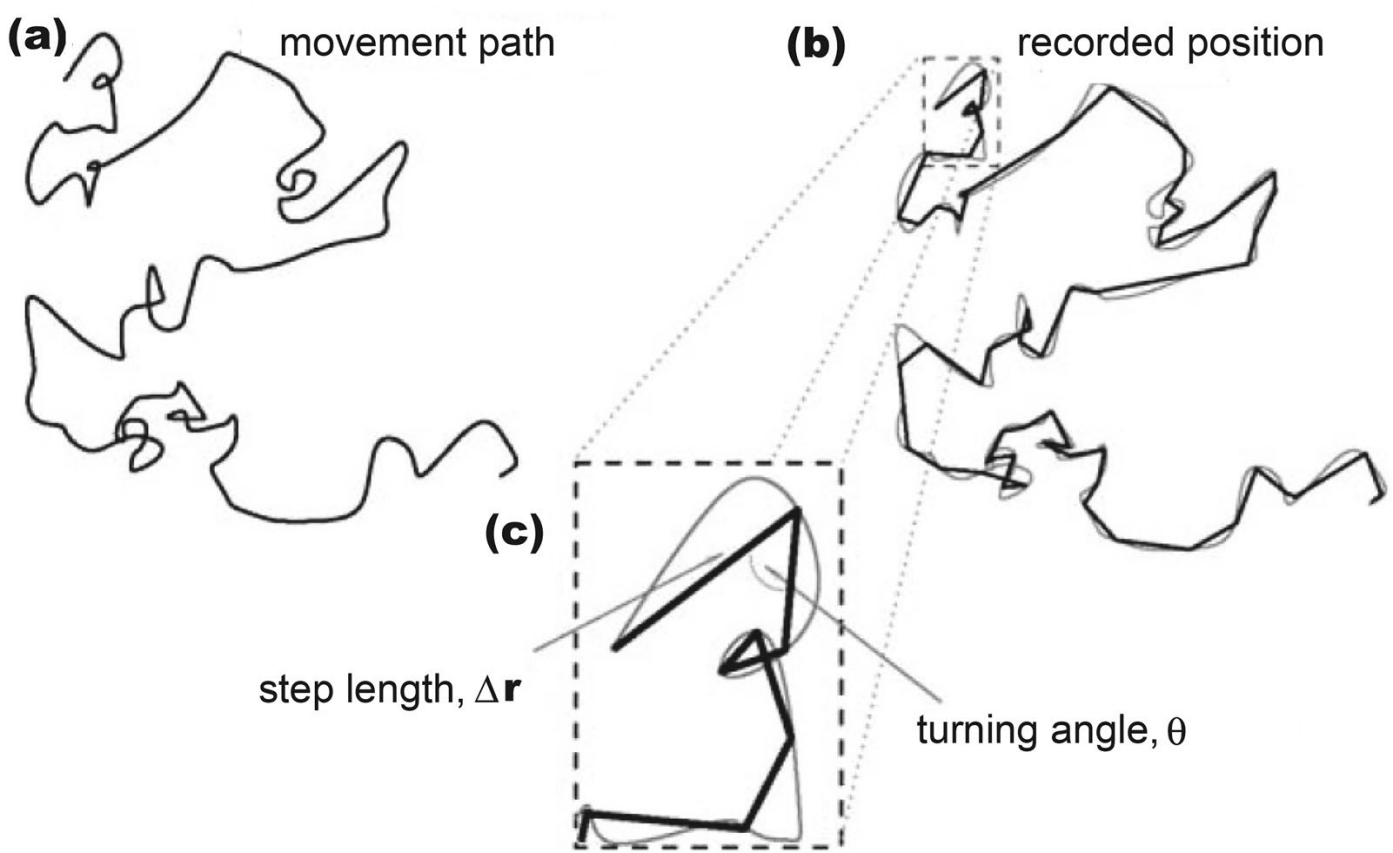
A sketch of animal movement path and its discretization (adapted from^36^). **(a)** The original movement path is normally curvilinear. **(b)** Due to the limitations of the radio-tracking technique, position of the animal is only known at certain discrete moments of time; correspondingly, the curve is approximated by a broken line. **(c)** The movement path as a broken line is fully described by the sequence of the step sizes (lengths) along the path, i.e. the distances travelled between any two sequential recorded positions, and the sequence of the corresponding turning angles.

The nature of the slug movement data collected in our field experiment (i.e. position on or beneath the soil surface observed at discrete moments of time) makes it possible to analyse the slug locomotory track in terms of the discrete-time random movement framework^12,16,35,36^. Within this framework, a curvilinear movement path is approximated by a broken line (see Fig. 2) and the movement of an individual slug is parameterized by the following frequency distributions:

1. The distribution of the step sizes along the movement path (i.e. the distance between sequential pairs of recorded positions; Fig. 2) or the corresponding average speed
2. The distribution of turning angle (the angle between the straight lines drawn between sequential pairs of recorded positions; Fig. 2).

Once all the information is available, it is possible to calculate the mean squared displacement as a function of time^12,37^. Additionally, in case the movement consists of alternating periods of active movement and immobility (periods with no recorded displacement resulting from feeding or inactivity, hereafter referred to as“resting time”), one should also consider the distribution of the corresponding periods.

### Speed, squared displacements and the straightness index

It is apparent from the data that slug movement is intermittent, with periods of locomotion interspersed between periods in which they remain motionless. Tables 1 and 2 show, for the sparse and dense releases respectively, the number of ‘active’ time intervals when the slugs were moving. Periods during which slugs were motionless are marked by the zeros in Tables 1 and 2, but all these individuals resumed their movement during the following hours, confirming that they were alive throughout the assessment period. We therefore retain the zeros in the data for the subsequent analysis.

**Table 1:** Slug mean speed (averaged over the whole movement path), the mean SSD (see Eqs. (3) and (5), respectively) and the straightness index in the case of sparse release for each of 17 slugs used in the experiment. Here the straightness index is calculated using Eq. (4) where the values of the step size are immediately available from our field data.

Slug number in protocol	Active steps (out of total 10)	Mean speed, $\langle v \rangle$	Mean SSD, $\langle \sigma ^2 \rangle$	Straightness index, *s*
1	4	0.0313	0.1681	0.7459
2	10	0.4731	14.6612	0.1758
3	8	0.1844	3.3475	0.7140
4	8	0.1781	3.0618	0.2231
5	7	0.3121	10.2142	0.7709
6	3	0.0389	0.2944	0.8337
7	2	0.0310	0.5091	0.8947
8	4	0.0670	0.7159	0.7055
9	6	0.4062	23.9861	0.1374
10	2	0.0400	0.4931	0.9088
11	2	0.0651	0.9406	1.0000
12	0	0	0	−
13	8	0.3719	12.9547	0.5115
14	6	0.1863	5.4126	0.4252
15	7	0.0847	0.9628	0.3668
16	3	0.0402	0.4149	0.4495
17	2	0.0774	1.4697	0.7685

**Table 2:** Slug mean speed (averaged over the whole movement path), the mean SSD (see Eqs. (3) and (5), respectively) and the straightness index in the case of dense release for each of 11 slugs used in the experiment. Here the straightness index is calculated using Eq. (4) where the values of the step size are immediately available from our field data.

Slug number in protocol	Active steps (out of total 10)	Mean speed, $\langle v \rangle$	Mean SSD, $\langle \sigma ^2 \rangle$	Straightness index, *s*
1	7	0.2946	12.4178	0.6825
2	0	0	0	−
3	4	0.0826	1.3006	0.9623
4	0	0	0	−
5	0	0	0	−
6	4	0.0887	1.4682	0.5321
7	3	0.0456	0.8170	0.4969
8	1	0.0222	0.3004	1
9	1	0.0734	2.9659	1
10	4	0.2137	5.6046	0.9965
11	2	0.0660	1.1160	0.9896

The baseline discrete-time framework considers animal position at equidistant moments of time. However, in the field experiment (as described in the previous section), time taken to locate slugs at each assessment resulted in the time interval varying between measurements (sparse release treatment: 27–87 mins; dense release treatment: 20–103 mins). The step size, i.e. the displacement during one time interval, depends in part on the duration of that interval, hence risking bias in the results. We address this issue by scaling the step size by the duration of the corresponding time interval, i.e. by considering the average speed during the step:

1 $$\begin{aligned} v_k(i)=\, & {} \frac{|\Delta {\mathbf{r}|_k(i)}}{\Delta {t}_k(i)}, \quad i=1,2,\ldots ,N, \end{aligned}$$

where

2 $$\begin{aligned} |\Delta {\mathbf{r}|_k(i)}=\, & {} |\mathbf{r}_k(t_i)-\mathbf{r}_k(t_{i-1})|, \end{aligned}$$

is the displacement of the *k*th slug during the *i*th time interval, i.e. the distance between the two sequential positions in the field. Here *N* is the total number of steps made by the given slug during the full period of the experiment (in our field data, for all slugs $N=10$).

For each individual slug, we then calculate the mean speed over all steps along the movement path:

3 $$\begin{aligned} <v>_k=\, & {} \frac{1}{N} \sum ^{N}_{i=1} v_k(i). \end{aligned}$$

**Figure 3:**
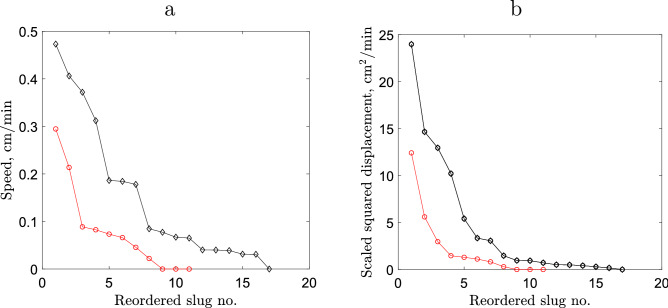
**(a)** Slug mean spead and **(b)** slug mean SSD, black diamonds for the sparse release and red circles for the dense release.

The results for the sparse and dense releases are shown in Tables 1 and 2, respectively; see also Fig. 3a.

The mean speed of slug movement, although being an important factor for slug dispersal, does not provide enough information about the rate at which the slug increases its linear distance from the point of release, because it does not provide information on the frequency of turning or the turning angle. In order to take that into account, we calculate the straightness index^35^, i.e. the ratio of the total displacement (distance between the point of release and the final position at the end of the experiment) to the total distance travelled along the path:

4 $$\begin{aligned} s_k= & {} |\mathbf{r}_k(t_N)-\mathbf{r}_k(t_0)|/\left( \sum ^{N}_{i=1}|\Delta {\mathbf{r}}|_k(i) \right) , \end{aligned}$$

where $t_0$ is the time of slug release and $t_N$ is the time of the final observation. The actual distance travelled is approximated by the length of the corresponding broken line (see the dark solid line in Fig. 2).

The straightness index quantifies the amount of turning (a combination of the frequency and angles of turns) along the whole movement path, i.e. over the whole observation time, but it says nothing about the rate of turning on the shorter time scale of a single ‘step’ along the movement path. To account for this, along with the mean speed we calculate the mean scaled squared displacement (SSD):

5 $$\begin{aligned} \langle \sigma ^2 \rangle _k=\, & {} \frac{1}{N} \sum ^{N}_{i=1} \sigma ^2_k(i) \qquad \text{ where }\qquad \sigma ^2_k(i)~=~\frac{|\Delta {\mathbf{r}|^2_k(i)}}{\Delta {t}_k(i)}, \end{aligned}$$

see Tables 1 and 2 and Fig. 3b. For the same value of mean speed, a larger value of the SSD corresponds to a straighter movement on the timescale of a single step, with a smaller turning rate.

An immediate observation from visual analysis of the data shown in Fig. 3 is that both slug speed and the SSD are smaller in the case of dense release than in the sparse release. Therefore, a preliminary conclusion can be drawn that average slug movement is slower in the dense release compared to the sparse release treatment.

### Turning angles

**Figure 4:**
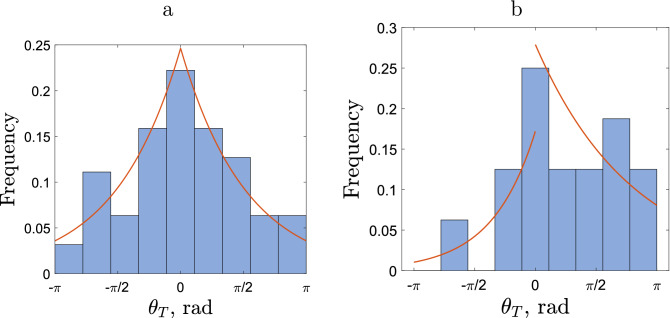
Frequency distribution of the turning angle in the case of **(a)** sparse and **(b)** dense releases of slugs. In calculating the turning angle, the periods of no movement were disregarded. The red curve shows the best-fitting of the data with the exponential function; see details in the text.

We now proceed to analyse the distribution of turning angles. The histogram of different values of the angle is shown in Fig. 4. Let us consider first the case of sparse release (see Fig. 4a). We readily observe that the distribution is roughly symmetrical and has a clear maximum at $\theta _T=0$. The latter indicates that, on this timescale, slug movement is better described as the CRW than the standard diffusion^1,16^. Indeed, the standard diffusion (also known as the simple random walk) assumes that there is no bias in the movement direction, in particular there is no correlation in the movement direction in the intervals before and after the recorded position, which means that the turning angle is uniformly distributed over the whole circle. On the contrary, in the case where a correlation between the movement directions exists (hence resulting in the CRW), the distribution of the turning angle becomes hump-shaped. This is in agreement with the results of previous studies on animal movement (in particular, invertebrates^12,38^) as well as a general theoretical argument^13^.

In order to provide a more quantitative insight, we look for a functional description of the turning angle distribution using several distributions that are commonly used in movement ecology. The results are shown in Table 3. We establish that the turning angle data are best described by the exponential distribution. Somewhat unexpectedly, it outperforms the Von Mises distribution, although the latter is often regarded as a benchmark and its use has some theoretical justification^1^. However, the exponential distribution of the turning angle has previously been observed in movement data on some other species, e.g. on swimming invertebrates^39^.

**Table 3:** The $r^2$ values for the turning angle movement data (in case of sparsely released slugs) described by different standard frequency distributions. The corresponding data are shown in Fig. 4a.

Distribution	Best fit	$r^2$
Uniform	0.1	$<0.001$
Piecewise linear	$0.205-0.0603|\theta |$	0.789
Von Mises	$\frac{0.672\exp (0.807 \cos (\theta ))}{2\pi I_0(0.807)}$	0.766
Power law	$36.1(4.28+|\theta |)^{-3.39}$	0.785
Exponential	$1.63\exp (-|\theta |/0.246)$	0.793

The distribution of turning angle obtained in the case of dense release exhibit different features; see Fig. 4b. However, in this case, the distribution is not symmetric and has a clear bias towards positive values: the mean turning angle corresponding to the data shown in Fig. 4b is $\langle \theta _T \rangle = 0.772\approx \pi /4$. Since the slugs used in the dense release are from the same cohort as those used in the sparse release, we consider this bias as an effect of the slug density: the movement pattern of an individual slug is affected by the presence of con-specifics. We discuss possible specific mechanisms for the responsiveness to this factor in the Discussion.

An attempt to describe the turning angle data from the dense release by a symmetric distribution returns low values of $r^2$ (see Suppl. Appendix A.1). However, the accuracy of data fitting comparable with the sparse release can be achieved by using an asymmetric distribution, i.e. where the corresponding function has different parameters for the positive and negative values of the angle. The results are shown in Table 4.

**Table 4:** The $r^2$ values for the turning angle movement data (in case of densely released slugs) described by asymmetric frequency distributions. In calculating the turning angle, the periods of no movement were disregarded; the corresponding data are shown in Fig. 4b.

Distribution	Best fit $\theta <0$	Best fit $\theta >0$	$r^2$
Uniform	0.05	0.15	0.348
Piecewise linear	$0.128+0.0448\theta$	$0.259-0.0627\theta$	0.750
Von Mises	$\frac{0.351\exp (1.19 \cos (\theta ))}{2\pi I_0(1.19)}$	$\frac{0.990\exp (0.491 \cos (\theta ))}{2\pi I_0(0.491)}$	0.707
Power law	$185(4.24+|\theta |)^{-4.78}$	$32.7(5.61+|\theta |)^{-2.74}$	0.727
Exponential	$0.172\exp (-|\theta |/1.12)$	$0.279\exp (-|\theta |/2.53)$	0.734

**Figure 5:**
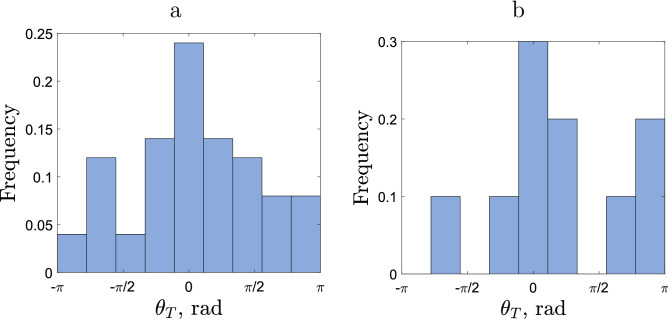
Frequency distribution of the turning angle in case of **(a)** sparse release, **(b)** dense release. The turning angle is only calculated for consecutive movements, i.e. if a slug does not move during a time step then its previous angle of movement is not used.

The turning angle data shown in Fig. 4 were obtained using all active steps along the movement paths. However, since periods of slug movement alternate with periods of resting, it may raise the question of the relevance of the turning angle at the locations where slugs remained motionless for some time. In order to check the robustness of our results, we now repeat the analysis to calculate the turning angle differently by omitting the segments adjoined with the rest position. The results are shown in Fig. 5. In this case, a reliable fit may not be possible due to there being insufficient data. However, a visual inspection of the corresponding histograms suggests that the main properties of the turning angle distribution agree with those observed above for the bigger data set. Namely, in both cases the distribution has a clear maximum at $\theta _T=0$ (this is seen particularly well in the case of sparse release). In the case of sparse release the distribution is approximately symmetric, while in the case of dense release there is a clear bias towards positive values. We therefore conclude that the properties of the turning angle distribution are robust with regard to the details of its definition.

### Movement and resting times

**Figure 6:**
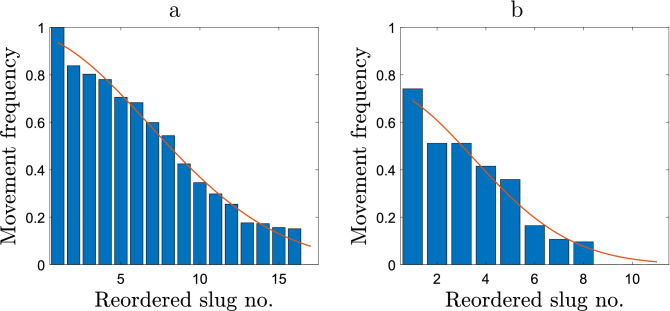
Distribution of the proportion of the total time spent in movement in case of **(a)** sparse release and **(b)** dense release. The red curve shows the best-fit of the data with the normal distribution; see details in the text.

Our field data shows that, while foraging, slugs do not move continuously but alternate periods of movement and rest; see the second column in Tables 1 and 2. Such behaviour is typical of many animal species^38,40^. In this section, we analyse the proportion of time that slugs spend moving, in particular to reveal the differences, if any, between the sparse and dense release.

Figure 6 shows the corresponding data where for the convenience of analysis the slugs are renumbered in a hierarchical order, so that slug 1 spends the highest proportion of time moving, slug 2 has the second highest, etc. We readily observe that the sparse release slugs tend to move more frequently than those from the dense release treatment: slugs that move for more than half of the total observation time constitute about 50% of the group in the case of sparse release but less than 30% in the case of dense release.

**Figure 7:**
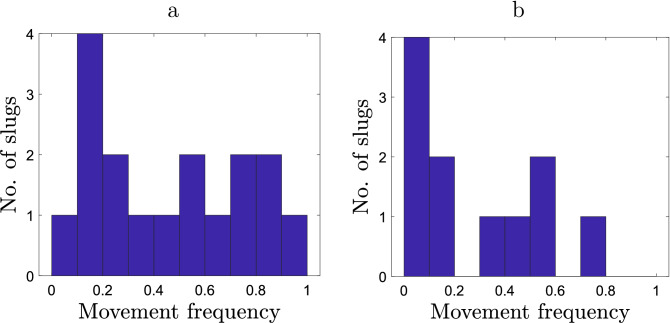
Distribution of the movement frequencies in case of **(a)** sparse release and **(b)** dense release.

In order to make a more quantitative insight, we endeavour to describe the data using several standard distributions; see Tables 5 and 6. We find that the normal distribution performs better than others both in sparse and dense release treatments. Importantly, however, the parameters of the distribution are significantly different between the two cases; in particular, the standard deviation appears to be approximately twice as large in the case of sparse release. Arguably, it confirms the above conclusion that slugs move more frequently or for longer in the case of sparse release. Slugs released as a group tend to spend considerably more time at rest compared to the slugs released individually.

**Table 5:** The $r^2$ values for the proportion of movement time described by different standard frequency distributions in the case of sparse release.

Distribution	Best fit	$r^2$
Linear	$0.994-0.0586x$	0.978
Normal	$3.21\frac{ \exp ( -0.5(x+1.47)^2/(8.21^2)) }{ 8.21\sqrt{2\pi }}$	0.983
Power law	$13900(20.3+x)^{-3.10}$	0.916
Exponential	$1.18\exp (\frac{-x}{8.37})$	0.945
Cauchy	$\frac{19.0}{6.65\pi } \frac{6.65^2}{ (x-1.56)^2 + 6.65^2 }$	0.962
Log-Cauchy	$\frac{13.4}{x\pi }\frac{1.07}{(\ln x-1.92)^2+1.07^2}$	0.977
Weibull	$8.89\frac{1.18}{7.25}\frac{x}{7.25}^{1.18-1} e^{(-(x/7.25)^{1.18}}$	0.971
Logistic	$17.4\frac{exp(-(x-0.189)/4.66)}{4.66( 1+exp( -(x-0.189)/4.66 ) )^2 }$	0.980

To avoid a possible bias due to the different group size (17 slugs in the sparse release and 11 in the dense release), we now rearrange the data in terms of the proportion of the group that moves with a given frequency. The results are shown in Fig. 7. Although the amount of data in this case does not allow us to describe them using a particular function, the two cases clearly exhibit distributions with different properties. In particular, the average movement frequency is 0.467 for the sparse release and 0.264 for the dense release, and the corresponding variances are 0.090 and 0.065, respectively.

**Table 6:** The $r^2$ values for the proportion of movement time described by different standard frequency distributions in the case of dense release.

Distribution	Best fit	$r^2$
Linear	$0.710-0.0743x$	0.930
Normal	$1.12\frac{ \exp ( -0.5(x+0.573)^2/(4.03^2)) }{ 4.03\sqrt{2\pi }}$	0.966
Power law	$42,500(12.1+x)^{-4.24}$	0.910
Exponential	$0.998\exp (\frac{-x}{3.68})$	0.933
Cauchy	$\frac{6.79}{3.12\pi } \frac{3.12^2}{ (x-0.932)^2 + 3.12^2 }$	0.925
Log-Cauchy	$\frac{5.22}{x\pi }\frac{1.13}{(\ln x-1.21)^2+1.13^2}$	0.928
Weibull	$3.30\frac{1.25}{3.53}\frac{x}{3.53}^{1.25-1} e^{(-(x/3.53)^{1.25}}$	0.953
Logistic	$6.46\frac{exp(-(x-0.232)/2.27)}{2.27( 1+exp( -(x-0.232)/2.27 ) )^2 }$	0.958

**Figure 8:**
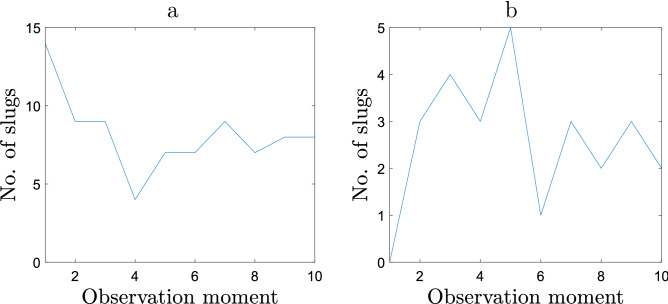
The number of moving slugs at each observation moment in case of **(a)** sparse release and **(b)** dense release.

To further quantify the differences, Fig. 8 shows the number of slugs moving in each observation interval. Once again, we observe that the graph exhibits essentially different properties between the two releases. In particular, over the first interval, the majority of slugs (14 out of 17) move in the case of sparse release but none of the slugs move in the case of dense release. In the second half of the observation time (intervals 6–10) on average about 50% of slugs (8 out of 17) move in the case of sparse release but only about 25% of slugs (2–3 out of 11) move in the case of dense release.

Based on the differences between the two releases, we conclude that the presence of con-specifics is the factor that affects the distribution of slug movement time. Thus, along with the results of the previous sections, it suggests that slug movement is density dependent.

## Rate of spread

It is a generic property of animal movement, especially during foraging, that they tend to move away from their original position—in our experiment, their release point. For many theoretical and practical reasons, assessment of the ‘rate of spread’ is often required (the rate at which the animals move away), e.g. by estimating the dependence of their Mean Squared Displacement (MSD) on time. In this section, we analyse the rate of spread using different approaches to confirm the above result that slugs move differently (and, on average, faster) in the sparse release than in the dense release.

### Mean squared displacement

**Figure 9:**
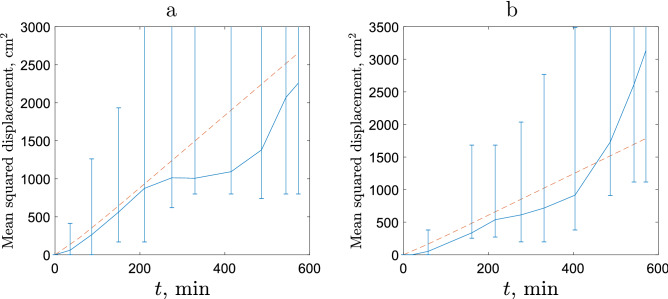
Mean Squared Displacement vs time averaged over all slugs in case of **(a)** sparse release and **(b)** dense release. Blue solid line shows the data, the red dashed line shows the theoretical prediction. The vertical lines show the actual range for the position of individual slugs from the field data.

Once sufficient data are available for the distribution of the turning angle and the displacement per unit time for an individual animal, its MSD can be predicted using the Correlated Random Walk (CRW) framework (see^12,16^ and Suppl. Appendix A.2). However, that would require a much larger amount of individual movement data than is available from our field experiment. We therefore have to pool the data from several individual slugs. We readily observe (Fig. 9) that the CRW predicts a faster rate of displacement in the case of sparse release. We also observe that the theoretical prediction (shown by the red line) is in good agreement with the data on the average slug displacement over the first 200 min of slug movement. However, the variance of the actual slug position grows rapidly with time: starting from approximately 150–200 min after release, it becomes so large that the prediction has little practical value.

One reason for the large variance of the calculated MSD is that there is considerable variability between the movement behaviour of different individual slugs, e.g. in terms of their mean speed (see Tables 1 and 2). In order to minimize the effect of individual differences while maintaining a sufficient volume of data for the analysis, we now quantify the rate of spread by analysing the data on the Scaled Squared Displacement (SSD) from slugs that show similar movement properties.

**Figure 10:**
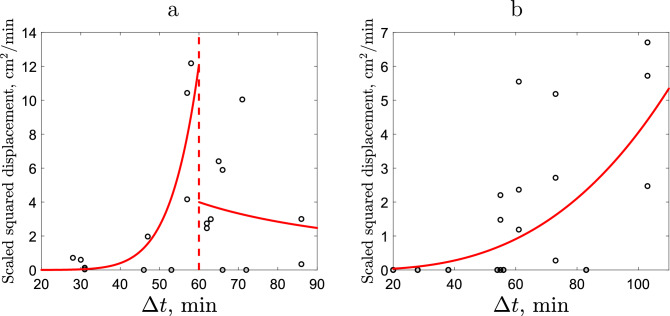
Values of the Scaled Squared Displacement vs time. **(a)** Data for slugs 3 and 4 in the sparse release (see Table 1) and its best fitting by the piecewise power law (red curve) with $1.01\times 10^{-16}\cdot (\Delta {t})^{9.62}$ for $27<\Delta {t}\le 60$ and $508\times (\Delta {t})^{-1.18}$ for $60<\Delta {t}\le 87$, $r^2=0.513$. **(b)** Data for slugs 3, 6 and 7 in the dense release (see Table 2) and its best fitting by the power law (red curve) with $5.81\times 10^{-6}\times (\Delta {t})^{2.92}$, $r^2=0.394$.

We begin with the case of sparse release. Figure 10a shows the cumulative data for the SSD for slugs 3 and 4, which have very similar values of mean speed (see Table 1). (We mention here that, where possible, one should avoid pooling together movement data of individual with different movement characteristics as such pooling may lead to unrealistic, superficial results, e.g. see^41^.) We readily observe that the ‘cloud’ of data points has a clear maximum at intermediate values of $\Delta {t}$. This does not fit into the standard theoretical framework that predicts the SSD to be a monotonously increasing function of time (see Suppl. Appendix A.2). It indicates that the SSD over the entire range cannot be linked to a single movement behaviour. We therefore assume that the slug movement occurs as a result of the interplay between two different movement behaviours, one prevailing at smaller time intervals (e.g. the CRW) and the other one prevailing at larger time intervals. Correspondingly, we endeavour to describe the SSD data by a piecewise function that is an increasing function at smaller time (on the left of the dashed vertical line) and a decreasing function at larger time (on the right of the dashed vertical line). Following established theory^1,42^ (see also Suppl. Appendix A.2), we describe the SSD by the power law.

The summary of the results (details of data fitting including the values of $r^2$ are given in Suppl. Appendix A.3) is shown in Fig. 10a. We readily observe that the SSD is a rapidly increasing function of time (much faster than linear), suggesting that slug movement on this short timescale can be classified as “superdiffusive” or even “super-ballistic”^42,43^. The CRW with the properties of superdiffusive movement has previously been observed for some other invertebrate species^44^. For larger time intervals, slugs movement slows down. Our results show that the dependence of the SSD on time is then well described by a power law with a negative exponent. Arguably, it suggests the “subdiffusive” movement pattern^42^. We hypothesize that this might be a manifestation of homing behaviour. Although evidence for homing behaviour in slugs is scarce^45,46^, it is well established in some other ground-dwelling invertebrates species, e.g. see^47^ and references therein. We will further discuss this issue in “Discussion”.

Interestingly, the data distribution of SSD in the case of dense release exhibits considerably different properties. Figure 10b shows the pooled data from slugs 3, 6 and 7. We readily observe that in this case the data are not peaked at the intermediate time but show a clear trend to increase. There is no indication of slowing down at the longer timescale and the best fitting is achieved by a simple power law (red curve) with the exponent larger than 2, thus suggesting superdiffusive movement over the entire observation time.

### Size and area of the patch

It is known that in agricultural environments (e.g arable fields), the spatial distribution of the grey field slug is heterogeneous, forming patches of high population density separated by areas with low population density^28,48^. In applications, e.g. for efficient pest management, it is important to understand how one can determine the patch location, how its boundary can be determined^24,25^, and how fast the patches evolve with time. There is considerable field evidence that patches of high slug density are stable in time, at least within a given season^28,48^. In addition, there are many theoretical results showing that density dependent individual movement is a factor that can increase patch stability and even lead to patch (cluster) formation^19,21,49,50^. Correspondingly, in this section we analyse the field data on slug movement in the context of patch dynamics.

The spatial ‘patch’ can be defined in several different ways. We notice here that, while a considerable proportion of the slug population is found in high-density patches, a (small) number of slugs can usually be found in the areas between the patches, although it remains unclear whether it is a result of a different movement behaviour (e.g. not directly controlled by the density dependence) or a random fluctuation. We therefore define the patch as the area that contains approximately 90% of the total number of slugs released in the given experiment.

**Figure 11:**
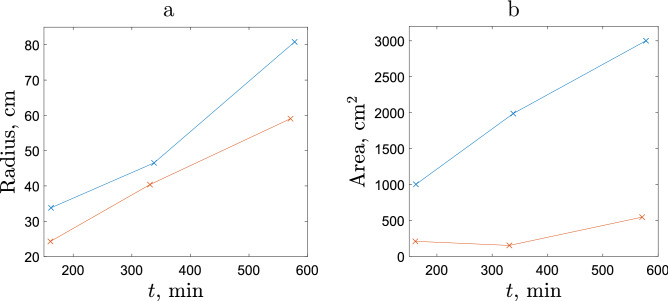
The radius **(a)** and the area **(b)** of the slug “patch” (cluster) shown after 4, 7 and 11 time steps as the time that the data was recorded are approximately the same for both sparse and dense releases. In **(a)**, the patch is defined as a circle that envelopes the closest 90% of slugs to the origin, its radius thus being the distance of its farthest slug. In **(b)**, in order to calculate the area the convex hull of the closest 90% of slugs was used. Blue and red colours are for the sparse and dense releases, respectively.

**Figure 12:**
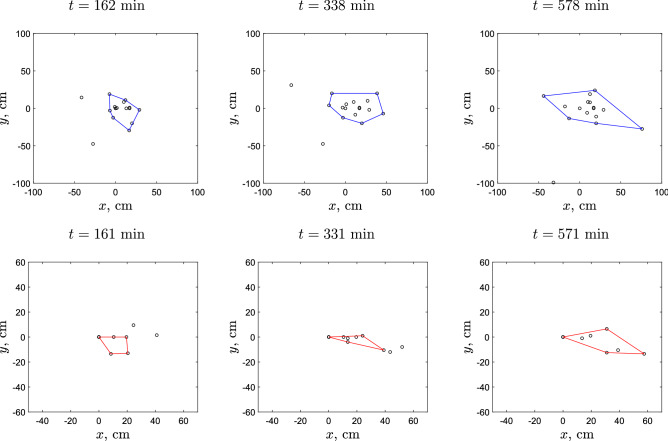
The slug patch (as defined by the convex hull that envelopes 90% of slugs closest to the origin) at different sequential time points after (top) sparse and (bottom) dense release. The time (in minutes) corresponds to time steps 4, 7 and 11 used in Fig. 11.

In the case of dense release, the above definition can be applied straightforwardly; see the red line in Fig. 11a. In sparse releases, the patch is virtual rather than real: in order to make the results comparable between the two releases, the start point for each individual movement path is translated to a common origin. The radius of the corresponding virtual patch vs time is shown in Fig. 11a by the blue line. We readily observe that the patch grows faster in the case of sparse release.

Apart from the size of the high density patch, as is quantified above by its radius, another important aspect is its shape. Indeed, the radius is not sufficient if the patch shape significantly deviates from the circle, e.g. is elongated: in this case, the same population can be spread over much larger distances. To take the effect of shape into account, arguably the area occupied by the patch should be considered along with the patch size.

In the light of the above, we analyse what is the patch shape and how it develops in time following sparse and dense releases. The results are shown in Figs. 11b and 12. We observe that the shape is similar in the two treatments. However, the difference between the two releases in terms of the area of the patch is clear, with the area growing considerably faster in the case of sparse release.

## Discussion

Good understanding of factors that determine the spatial distribution of invertebrates in their natural environment is needed for various purposes, for instance, conservation of endangered species and control of pest species. Density dependent individual movement is one such factor as it is known to contribute to the formation of pronounced spatial heterogeneity and to the temporal stability (i.e. persistence over long periods of time) of patches of high population density^19,20,50–53^. The existence of density related responses, although obvious in some species (e.g. through the formation of swarms) may be difficult to detect in other species. This study is motivated by the observation that slugs in agricultural environments often create patches of high population density even where the environmental properties (e.g. temperature, moisture and soil properties) are apparently uniform^28,29^. Arguably, in the absence of physical forcing, the only alternative is forcing through biological responses. Correspondingly, here our goal was to investigate the effect of density dependence on the movement of the grey field slug.

Slugs were radio-tagged and their movement tracked in a field experiment with two different treatments, i.e. following release as either a group (dense release) or with individuals placed far away from each other (sparse release) so that they were unlikely to encounter each other for the duration of the experiment. Slug movement data were analysed using the discrete-time random walk framework^1,16,35,54^ that parameterizes the movement with frequency distributions for its three essential components: the step size, the turning angle, and the proportion of movement/resting time. We mention here that the assumption of the randomness of the animal movement is context-specific; in particular, it depends on the type of movement. For instance, animal movement during their migration can hardly be regarded as random. However, animal movement during foraging can often be treated as random (e.g. see^11,16^) and described accordingly, i.e. by considering the constituting elements of the movement path as random variables described by certain probability distributions.

Our analysis reveals that the properties of all three movement components are significantly different between the sparse and dense releases, with the general tendency that slugs move faster and longer distances in the case of sparse release, i.e. in the absence of the con-specifics in their vicinity. We have further confirmed it by estimating the rate at which the ‘patch’ (the area containing 90% of the group) grows with time. Consistently with the above, we obtained that the patch grows slower in the case of dense release. Arguably, the latter has an immediate interpretation that the density dependence of the individual slug movement enhances the stability of slug patches.

A question may arise here as to why slugs move faster and longer distances in the case of sparse release. As a probable answer, the reduced encounter rate with conspecifics or signs of conspecifics (e.g. chemical signals, slime trails, etc.) may result in movement being interrupted less often and contribute to a lower turning frequency (the latter linearising the track), in combination resulting in a greater rate of displacement. This may result in more rapid displacement between than within patches.

**Figure 13:**
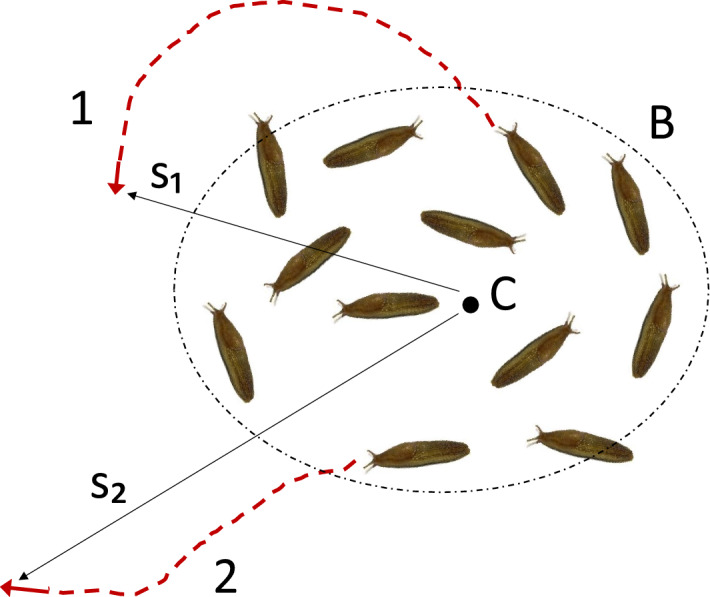
Sketch of the effect of animal movement path’s geometry on the patch temporal stability. Here point *C* is the centre of the patch, *B* is the hypothetical patch boundary, lines 1 and 2 are two movement paths with different properties, and $s_1$ and $s_2$ are the distance from the patch centre after a given time. For approximately the same distance travelled along the path, a slug that moves along a spiral-like path (line 1) will on average remain significantly closer to the patch centre (note $s_1<s_2$) and/or to the patch boundary than a slug that moves along an approximately straight line (line 2).

One of the interesting findings of our study is the strongly biased distribution of the turning angles observed in the case of dense release. Although our data do not allow for the identification of the corresponding movement behaviour, candidate contributory mechanisms are known from the literature. It has previously been shown that *D. reticulatum* is able to re-locate refuges from distances of 1m or even larger^46^, and that some slug species (e.g. the pulmonate slug *Limax pseudoflavus*) follow the slime trails of their con-specifics^55^. The use of chemical information has also been shown to influence homing in two *Limax spp.*^45,56^. In the case of *D. reticulatum* trail following has been recorded but occurs less frequently (8% of trail encounters) and is only exhibited when reproductively active, a small part of their lifetime^30^. Our results, however, indicate that, in spite of its relatively low frequency, the slime trail following, and possibly chemical signals, may be factors contributing to the turning angle bias. Indeed, in the case of dense release, fresh trails are abundant within a small area and even a relatively small proportion of the tailgating slugs can result in a noticeable bias in the turning angle distribution. Note that the persistent bias in the turning angle suggests that the corresponding movement paths are spiral-like. This, in turn, may result in increased cohesion of high density patches: indeed, a slug moving along a spiral would remain in the vicinity of the patch for a much longer time (see Fig. 13).

We have also shown that the presence of con-specifics has a more subtle effect on the individual movement by changing the way in which the SSD depends on the duration of the movement interval: the SSD is a monotonously increasing function of time in the case of dense release but a non-monotonous hump-shaped function in the case of sparse release. This may indicate that, in the absence of con-specifics, the individual slug movement behaviour is timescale dependent.

On the small timescale, slug movement occurs faster than is predicted by the standard Brownian (diffusive) motion. The question however remains whether this faster movement occurs because of the correlation between subsequent steps as described by the CRW or the movement is of a different type, i.e. superdiffusive or even superballistic (see Eqs. (1–2) in the Suppl. Appendix A.2). To look into this question, we compare the quality of data fitting provided by the power law with different exponents. Recall that, while the range of exponent values for the SSD dependence on time is the same for the CRW and for the superdiffusive movement (cf. Eq. (1) for $1<\gamma <2$ with Eqs. (4) and (6) in the Suppl. Appendix A.2), for the superballistic movement the exponent is larger than 1. Our results (see Fig. 10 and Suppl. Appendix A.2 for more details) show that the exponent is consistently larger than 2, which leads us to the conclusion that, on the shorter time scale, slugs perform superballistic movement.

We mention here that the above conclusion about the superballistic movement (often referred to in the literature as the Lévy walk) is in good agreement with some other studies on invertebrates. In particular, the Levy walk type movement was observed in mud snails^57^ and Drosophila larvae^58^. Interestingly, a study on the movement behavior of walking Tenebrio beetles^44^, while observing a superballistic movement, concluded that the correlated random walk can produce a movement pattern indistinguishable from the Levy walk. An unambiguous identification of the movement pattern requires the movement data from multiple temporal scales as the pattern can be scale dependent, e.g. being superballistic at small scales but slowing down to diffusion at large temporal scales^59,60^. Such slowing down can happen due to different mechanisms, e.g. as a behavioural response to meeting con-specifics^59^ or due to the inherent effect of the environmental friction^60^. Here we hypothesize that slowing down of slug displacement (as observed in the case of the sparse release, see Fig. 10a) can also result from the homing behaviour. Alternatively, a well developed theory^61^ in agreement with some empirical studies^37,62^ predicts that the Levy walk type movement can arise as the asymptotical regime of the composite random walk when the spatial scales involved are sufficiently broad to allow for the excitation of a large number of elementary movement modes. Therefore, although our study indicates the superballistic movement of slugs at a small timescale (one day), further investigation is needed to reveal the movement properties on a larger timescale (e.g. weeks or months).

In summary, using a novel combination of mathematical and biological techniques to investigate the effect of slug population density on their locomotory behaviour, in our study we have found that all components of the slug movement (mean speed, turning angles and movement/resting times) exhibit significantly different properties in the cases of sparse and dense releases. As a result, the slugs released as a group dispersed more slowly than those released individually In particular, the turning angle of those released as a group (dense release) displayed a clear anticlockwise bias. This clearly suggests that the density is a factor regulating slug movement in the agricultural environment. While the dense release included eleven tagged individuals, however, there was only one group. Further work is therefore required to confirm these preliminary findings, which may provide an insight into the behavioural mechanisms underpinning the stability of the high density patches that are a feature of slug recognized heterogenous distribution in arable fields. This would facilitate the targeting of pesticide applications to discrete areas of the field, thus providing an approach to more sustainable slug control.

## Supplementary information

Supplementary Information
