# Supplementary material for: Movement patterns of the grey field slug (Deroceras reticulatum) in an arable field
Source: Sci Rep. 2020 Oct 21;10:17970. doi: 10.1038/s41598-020-74643-3 (PMC7578660; doi:10.1038/s41598-020-74643-3)
Supplement: Supplementary file 1 — Supplementary Information [file 41598_2020_74643_MOESM1_ESM.pdf]

Movement Patterns of the Grey Field Slug (*Deroceras reticulatum*) in an Arable Field

J. Ellis, N. Petrovskaya, E. Forbes, K.F.A. Walters, S. Petrovskii

A Supplementary Material

A.1 Turning angle frequency

Table A.1 shows the results of fitting the distribution of the turning angle data from the dense release by a symmetric distribution. One readily observes that the values of r^2 are considerably less than those obtained in the case of using an asymmetric distribution; see Table 4 in the main text.

Table A.1: Examples of distributions fitted to the turning angle of all densely released slugs, excluding no movements.

Distribution	Best fit	r^2
Uniform	0.1	2.22×10^{-16}
Piecewise Linear	$0.193 - 0.0537\|\theta\|$	0.391
Von Mises	$\frac{0.670\exp(0.658\cos(\theta))}{2\pi I_0(0.658)}$	0.351
Power Law	$36.0(4.85+\|\theta\|)^{-3.20}$	0.370
Exponential	$1.92\exp(-\|\theta\|/0.220)$	0.376

A.2 MSD and SSD

When analysing patterns of individual animal movement, a key question is how far the animals can spread over a given duration of time. In case of a random movement (see [4] for the discussion of the "bugbear of randomness"), the rate of spread is conventionally quantified by the dependence of the Mean Squared Displacement (MSD) on time. A well established theory [1, 4, 5] suggests that it should follow the power law, which we scale by the duration of the corresponding time interval, hence to obtain the Scaled Squared Displacement (SSD):

$$\frac{|\Delta\mathbf{r}|^2}{\Delta t} \sim (\Delta t)^{\gamma-1}, \tag{A.1}$$

Table A.2: A piecewise power law function fitted to $(\Delta x)^2/\Delta t$ plotted against Δt for sparsely released slugs 3 and 4. The two values of r^2 are for each section of the fit.

α	$x < \alpha$	$x > \alpha$	r^2
55	$0.00633x^{0.566}$	$4.32 \times 10^8 x^{-4.41}$	0.012, 0.248
57	$1.10 \times 10^{-14} x^{8.40}$	$1.67 \times 10^{10} x^{-5.28}$	0.623, 0.215
60	$1.01 \times 10^{-16} x^{9.62}$	$508x^{-1.18}$	0.754, 0.026
63	$8.20 \times 10^{-5} x^{2.68}$	$1.43 \times 10^6 x^{-3.01}$	0.263, 0.103
65	$6.09 \times 10^{-5} x^{2.76}$	$4.51 \times 10^4 x^{-2.22}$	0.303, 0.049
70	$8.30 \times 10^{-4} x^{2.08}$	$8.43 \times 10^{12} x^{-6.58}$	0.214, 0.243

where the exponent γ depends on the movement type, i.e. $\gamma = 1$ in case of the diffusive movement [2, 4] and $\gamma > 1$ in case of a faster movement which is often referred to as "superdiffusive" if $1 < \gamma < 2$, ballistic if $\gamma = 2$ and "superballistic" if $\gamma > 2$. The case of slower spread with $0 < \gamma < 1$ is called the "subdiffusive" movement.

It is useful to comment on the geometry of the corresponding graph. It is readily seen that the graph of relation (A.1) is given by a straight line for the ballistic case $\gamma = 2$, a convex curve for the superdiffusive case $1 < \gamma < 2$ and a concave curve for the superballistic if $\gamma > 2$.

Figure A.1 shows the results of fitting the values of SSD obtained in the case of sparse release by a piecewise power law subject to a different choice of the junction point. The corresponding values of r^2 are shown in Table A.2.

Note that strictly speaking relation (A.1) is only valid in an idealized case where the turning angle is distributed uniformly over the circle, i.e. there is no correlation between any two consequent movements along the path. A more realistic case is given by the Correlated Random Walk (CRW) where the distribution of the turning angle is lumped around the movement direction during the preceding interval [3]. In the case of equidistant observation moments (i.e. a constant time step), the dependence of the MSD on the number of steps n along the movement path is given by the following equation [3]:

$$|\Delta \mathbf{r}|^2(t) \quad = \quad \langle l^2 \rangle n + \frac{2c\langle l \rangle^2}{1-c}\left(n - \frac{1-c^n}{1-c}\right) \qquad \text{where} \qquad n = \frac{t}{(\Delta t)_0}. \tag{A.2}$$

Here $(\Delta t)_0$ is the duration of (fixed) time step and $\langle l \rangle$ and $\langle l^2 \rangle$ are, respectively, the mean and the variance of the step size distribution (assuming that they exist, which implies a thin-tailed distribution of the step size [1]) and c is the mean value of the cosine of the turning angle. In case the turning angle is distributed uniformly over the circle, i.e. $c = 0$ and expression (A.2) turns into

$$|\Delta \mathbf{r}|^2(t) \quad = \quad \langle l^2 \rangle n \ \sim \ t, \tag{A.3}$$

which corresponds to $\gamma = 1$ in (A.1) and hence to the case of diffusive spread.

In the general case $c \neq 0$, expression (A.2) describes the movement that in the course of time slows down from the almost ballistic movement $|\Delta \mathbf{r}|^2 \sim t^2$ to the diffusion motion

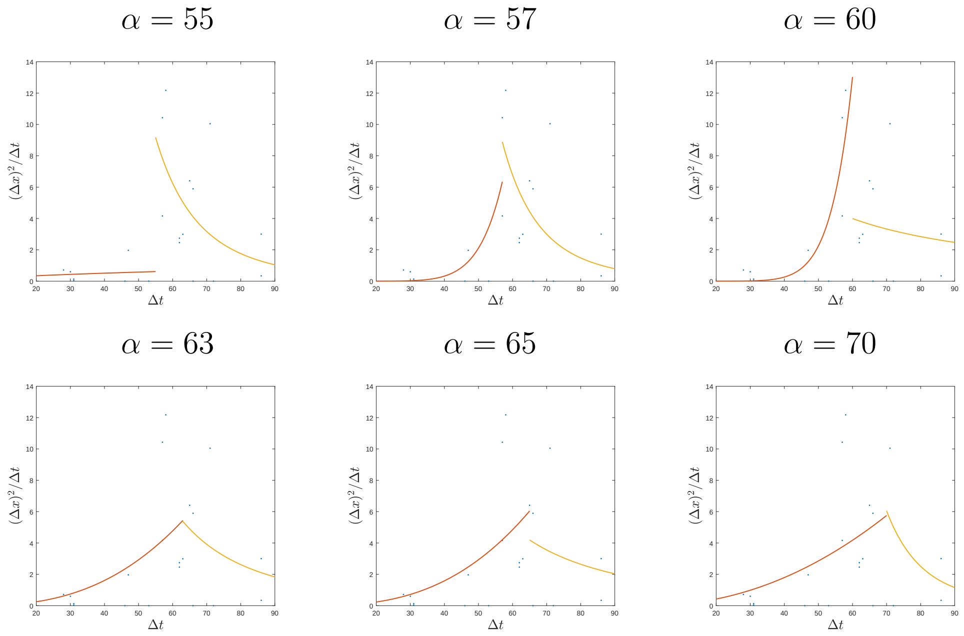

Figure A.1: $(\Delta x)^2/\Delta t$ plotted against Δt for sparsely released slugs 3 and 4, fitted with a piecewise power law function which is split at α.

$|\Delta \mathbf{r}|^2 \sim t$; see Ref. [1]. Indeed, it is readily seen that

$$|\Delta \mathbf{r}|^2 = \langle l^2 \rangle n + \frac{2c\langle l \rangle^2}{1-c} \sim n \sim t, \tag{A.4}$$

for a large number of steps n, and therefore the walk becomes diffusive in the long term. In order to obtain the expression for small number of steps, for the sake of simplicity let us consider the case with a high directional persistence, so that $c = 1 - \delta$ where $\delta \ll 1$. Then

$$1 - c^n = 1 - (1-\delta)^n \approx n\delta - \frac{1}{2}n(n-1)\delta^2 \tag{A.5}$$

(omitting terms containing higher orders of δ) so that Eq. (A.2) becomes

$$|\Delta \mathbf{r}|^2 = n\langle l^2 \rangle + \langle l \rangle^2 (1-\delta) n(n-1) \sim n^2 \sim t^2. \tag{A.6}$$

Therefore, the graph of the SSD in the case where the animal performs the CRW is also given by a concave curve but with slopes different from the one predicted by Eq. (A.1). Thus, should one of them provide a better description of the data than the other one, that should allow us to identify the corresponding movement pattern, e.g. CRW vs superballistic. However, the concavity of the graph may be difficult to observe if c is not close to one.

A.3 Movement and rest times

Figures A.2 and A.3 show, for the sparse and dense release respectively, the description of the data on movement/rest time with several standard distributions. Interestingly, although

the visual inspection of the quality of the data fit may favour either the logistic or the log-Cauchy distribution, a more quantitative analysis based on r^2 criterium shows that the normal distribution performs better than others; see details in Tables 5 and 6 in the main text.

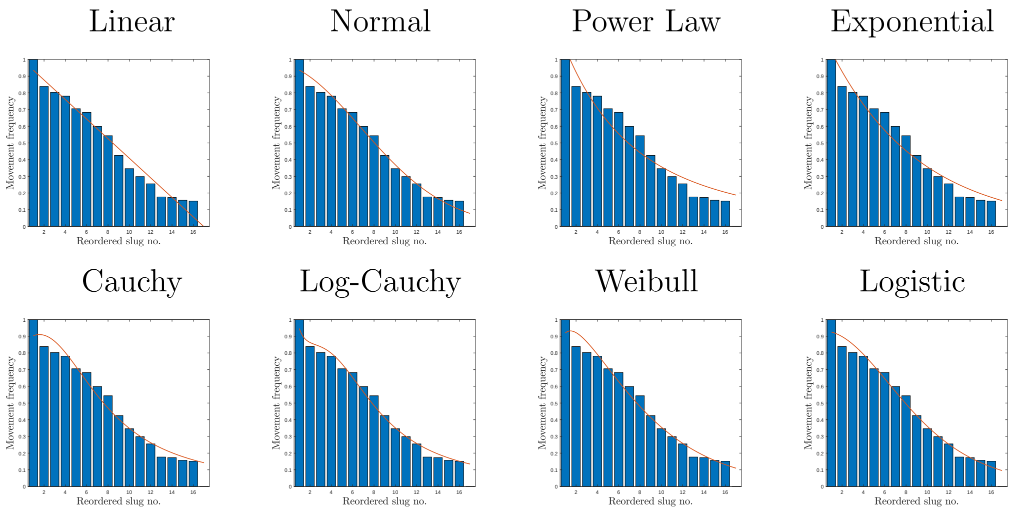

Figure A.2: Distribution of the proportion of the total time spent in movement in the case of sparse release. The red curve shows the best-fitting of the data with various standard distributions (as in the figure legend). The corresponding values of r^2 (quantifying the quality of fit) are shown in Table 5 in the main text.

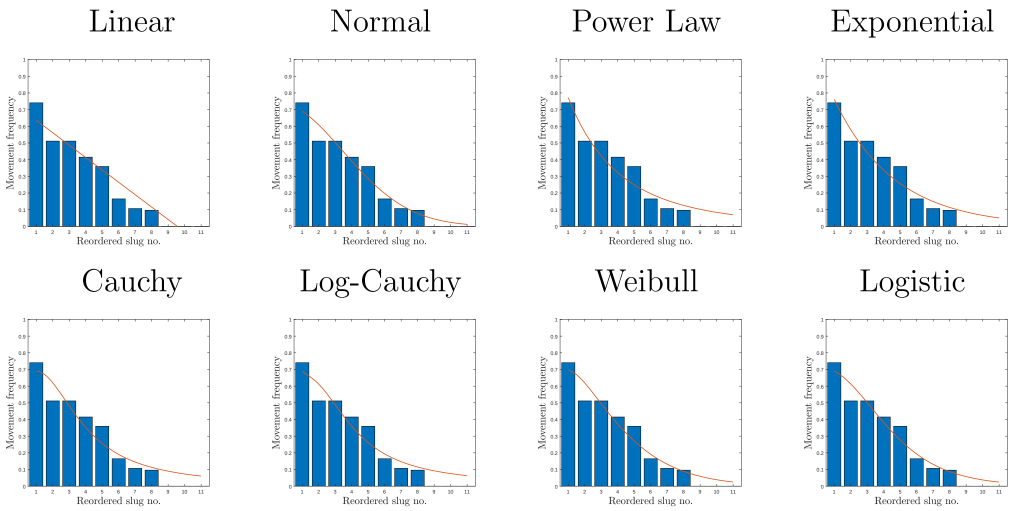

Figure A.3: Distribution of the proportion of the total time spent in movement in the case of dense release. The red curve shows the best-fitting of the data with various standard distributions (as in the figure legend). The corresponding values of r^2 (quantifying the quality of fit) are shown in Table 6 in the main text.

References

[1] Codling, E.A., Plank, M.J., Benhamou, S. (2008) Random walk models in biology. Journal of the Royal Society Interface, 5, 813-834.

[2] Kareiva, P.M. (1983) Local movement in herbivorous insecta: applying a passive diffusion model to mark-recapture field experiments. Oecologia, 57, 322-327.

[3] Kareiva, P.M., Shigesada, N. (1983) Analyzing animal movement as a correlated random walk. Oecologia 56, 234-238.

[4] Turchin, P. (1998) Quantitative analysis of movement. Sunderland, Sinauer.

[5] Viswanathan, G. M., da Luz, M. G. E., Raposo, E. P. & Stanley, H. E. (2011) The physics of foraging: an introduction to random searches and biological encounters. Cambridge University Press, Cambridge.
